# Genome-Wide Association Study and Genomic Prediction Elucidate the Distinct Genetic Architecture of Aluminum and Proton Tolerance in *Arabidopsis thaliana*

**DOI:** 10.3389/fpls.2020.00405

**Published:** 2020-04-09

**Authors:** Yuki Nakano, Kazutaka Kusunoki, Owen A. Hoekenga, Keisuke Tanaka, Satoshi Iuchi, Yoichi Sakata, Masatomo Kobayashi, Yoshiharu Y. Yamamoto, Hiroyuki Koyama, Yuriko Kobayashi

**Affiliations:** ^1^Faculty of Applied Biological Sciences, Gifu University, Gifu, Japan; ^2^Cayuga Genetics Consulting Group LLC, Ithaca, NY, United States; ^3^NODAI Genome Research Center, Tokyo University of Agriculture, Tokyo, Japan; ^4^Experimental Plant Division, RIKEN BioResource Research Center, Tsukuba, Japan; ^5^Department of Bioscience, Tokyo University of Agriculture, Tokyo, Japan

**Keywords:** acid soil tolerance, ALMT1, aluminum and proton tolerance, co-expression network analysis, ELP – expression level polymorphism, GP – genomic prediction, GWAS – genome-wide association study, natural variation

## Abstract

Under acid soil conditions, Al stress and proton stress can occur, reducing root growth and function. However, these stressors are distinct, and tolerance to each is governed by multiple physiological processes. To better understand the genes that underlie these coincidental but experimentally separable stresses, a genome-wide association study (GWAS) and genomic prediction (GP) models were created for approximately 200 diverse *Arabidopsis thaliana* accessions. GWAS and genomic prediction identified 140/160 SNPs associated with Al and proton tolerance, respectively, which explained approximately 70% of the variance observed. Reverse genetics of the genes in loci identified novel Al and proton tolerance genes, including *TON1-RECRUITING MOTIF 28* (*AtTRM28*) and *THIOREDOXIN H-TYPE 1* (*AtTRX1*), as well as genes known to be associated with tolerance, such as the Al-activated malate transporter, *AtALMT1*. Additionally, variation in Al tolerance was partially explained by expression level polymorphisms of *AtALMT1* and *AtTRX1* caused by cis-regulatory allelic variation. These results suggest that we successfully identified the loci that regulate Al and proton tolerance. Furthermore, very small numbers of loci were shared by Al and proton tolerance as determined by the GWAS. There were substantial differences between the phenotype predicted by genomic prediction and the observed phenotype for Al tolerance. This suggested that the GWAS-undetectable genetic factors (e.g., rare-allele mutations) contributing to the variation of tolerance were more important for Al tolerance than for proton tolerance. This study provides important new insights into the genetic architecture that produces variation in the tolerance of acid soil.

## Introduction

Acid soil syndrome is a serious limiting factor for food production worldwide (von Uexküll and Mutert, 1995; Kochian et al., 2004). In acid soil, plant root growth is inhibited by various stressors, such as rhizotoxicities of excess Al, proton, manganese (Mn), and iron (Fe), and deficiencies in the available phosphate (Pi) (Kochian et al., 2004). Plants have adapted to acidic environments by developing a number of stress tolerance mechanisms which can have pleiotropic effects on other traits. For example, organic acid excretion from roots contributes to Al tolerance and efficient P-utilization under acid soil conditions (see review, Wu et al., 2018). In contrast, the expression of Al and proton tolerance genes is co-regulated under the downstream of STOP1 (SENSITIVE TO PROTON RHIZOTOXICITY1) in *Arabidopsis thaliana* (Iuchi et al., 2007). Identification of the molecular mechanisms which underlie tolerance to co-existing stress factors may allow for improved crop yields in acid soils, through the use of biotechnology and molecular breeding.

Al occurs in many chemical forms in the soil but the higher concentration of soluble Al^3+^ cations that are present in acidic soils is a major limitation to many crop species. Al toxicity in the growing root tip is reversible over short periods of time, but over long-term exposure, Al treatment disturb various cellular processes, such as cell wall expansion and membrane transport (Ma, 2007). Molecular physiological studies of these events have identified a number of Al tolerance genes (see review, Kochian et al., 2015), and several Al tolerant transgenic crops have already been developed through the overexpression of Al tolerant genes. Ectopic expression of *ALUMINUM ACTIVATED MALATE TRANSPORTER 1* (*ALMT1*) from wheat (*Triticum aestivum*; Sasaki et al., 2004) in barley (*Hordeum vulgare*) (Delhaize et al., 2004), and of *CITRATE SYNTHASE* of *Arabidopsis* (*Arabidopsis thaliana*) in canola (*Brassica napus*) (Anoop, 2003) conferred Al tolerance. However, proton rhizotoxicity can be more toxic than Al rhizotoxicity in natural acid soils (Kinraide, 2003), and is also a complex polygenic trait which requires many genes to achieve distinct physiological processes (Shavrukov and Hirai, 2016). For example, the maintenance of cellular pH, which is essential for adapting to proton stress (Sawaki et al., 2009; Bissoli et al., 2012; Gujas et al., 2012) and the stabilization of pectin, which is essential for protection against proton toxicity (Koyama et al., 2001), are processes regulated by multiple genes. Identification of proton tolerance mechanisms and their interactions with Al tolerance is important for improving the acid soil tolerance of crops.

In certain plant species such as *Arabidopsis* and tobacco (*Nicotiana tabacum*), both Al tolerance and proton tolerance are mutually regulated by the STOP1 (SENSITIVE TO PROTON RHIZOTOXICITY 1) transcription factor (e.g., AtSTOP1, Iuchi et al., 2007, NtSTOP1, Ohyama et al., 2013). Al tolerance genes such as Al activated organic acid transporters (i.e., ALMT and MATE; see review, Daspute et al., 2017), and proton tolerance genes such as *AKT1*, *HAK5*, and *SULTR3;5* are co-regulated by STOP1 (Sawaki et al., 2009). In addition, activation of STOP1/ALMT1 is also involved in the low-phosphate response in *Arabidopsis*, and has been shown to alter root architecture to induce efficient P-uptake (Balzergue et al., 2017). These findings suggest that Al and proton tolerance are controlled by a common molecular mechanism. However, Al and proton tolerant mechanisms are complex and likely involve unidentified mechanisms. Elucidation of such complex adaptive mechanisms can be investigated using genome-wide approaches in *Arabidopsis* (*Arabidopsis thaliana*), that utilize differences in Al and proton tolerance among accessions.

Studies of the natural phenotypic variation in *Arabidopsis* may provide an opportunity to study interactions among Al and proton tolerance mechanisms, which usually co-exist in naturally acid soil environments (Ikka et al., 2007). A genome-wide association study (GWAS) in *Arabidopsis* is a useful way to clarify complex mechanisms, especially when integrated with other genomics approaches. Although a GWAS may likely yield poor detection of quantitative traits with weak locus effects (Bergelson and Roux, 2010), integration with other genome-wide approaches would help to clarify such effects included in the natural variation. For example, genomic prediction (GP), a genome-wide population genetic method, may allow for the assessment of the cumulative effects of associated loci (Crossa et al., 2010; Desta and Ortiz, 2014). Furthermore, integration of GP and co-expression gene network analysis could further improve the sensitivity and accuracy of the population genetic methods used for GWAS (Kobayashi et al., 2016; Kooke et al., 2016; Butardo et al., 2017). Novel and unidentified Al tolerance genes were detected previously using genome-wide expression level polymorphism [Expression level polymorphism (ELP); Delker and Quint, 2011] analyses, by comparing the transcriptomes of three Al tolerant and Al sensitive accessions (Kusunoki et al., 2017). This identified genes that had not previously been reported to relate to known Al tolerant mechanisms (e.g., Al extrusion and internal Al tolerant mechanisms), for example BINDING PROTEIN 3, that is linked to the quality control of proteins in endoplasmic reticulum. Integration of these approaches is a useful method to investigate the molecular determinants driving Al and proton tolerance mechanisms in plants. In this study, we conducted GWAS for Al and proton tolerance, and identified 140 and 160 loci respectively that explained approximately 70% of the variations estimated by GP. Application of other genome-wide approaches identified distinct Al and proton tolerance mechanisms, which independently segregated under natural conditions.

## Materials and Methods

### Plant Materials

Worldwide *Arabidopsis thaliana* accessions described in the 1001 Genomes Project^1^ were derived from the Arabidopsis Biological Resource Center, Nottingham Arabidopsis Stock Centre (NASC; Nottingham, United Kingdom), and RIKEN BioResource Research Center (RIKEN BR; Tsukuba, Japan) (Supplementary Table S1). The seed progenies used were obtained via single seed descent from the original seeds. Mutants and T-DNA/transposon insertion lines were obtained from NASC (Supplementary Table S2).

Transgenic Col-0 for GUS (β-glucuronidase) reporter assays used to characterize *AtALMT1* promoters were generated using *Agrobacterium tumefaciens* (GV3101)-mediated transformation, using methods described by Clough and Bent (1998). Promoter-GUS was cloned into the binary vector (pBE2113) by overlap-extension PCR (Horton et al., 1989) using the gene-specific primers described in Supplementary Table S3.

### Plant Growth Conditions and Phenotyping of Al and Proton Tolerance

Al and proton tolerance of accessions was judged by the relative root growth (treatments/control) of hydroponically grown seedlings as described previously (Kobayashi et al., 2007). Approximately 20 seedlings of accessions were grown hydroponically for 5 days in modified MGRL nutrient solution (Fujiwara et al., 1992), which contained 2% MGRL nutrients [other than P and Ca (-P, CaCl_2_ adjusted to 200 μM)]. The initial pH of the control (modified MGRL; no Al) and Al toxic (modified MGRL plus 5 μM of AlCl_3_) solutions were adjusted to 5.0, whereas that of the proton toxic (modified MGRL) solution was adjusted to 4.6. All solutions were renewed every two days. Approximately 200 accessions were equally divided into two and grown in each plastic container containing 10 L of culture solution using the method developed by Toda et al. (1999). The growth test at one condition among three conditions was conducted at the same time. Seedlings were placed on solidified agar (1%, w/v) and photographed using a digital camera (Canon EOS kiss X5). The length of the primary roots was then determined using LIA32 software (LIA for Win32^2^). Relative root length (RRL; root length under stressed conditions/root length under control conditions [%]) was calculated for each line using the five longest roots in each condition (average of five biological replicates seedlings, *n* = 5). All growth experiments were conducted under controlled environmental conditions (12 h day/night cycle, 37 μmol m^–2^ s^–1^ at 24°C ± 2°C). After removing accessions with low germination percentages, we obtained the phenotype of 206, 196, and 200 accessions under the control, Al stress, and proton tress conditions respectively. Broad-sense heritability (H_b_^2^) and CV were calculated following the methods of Ikka et al. (2007).

### Estimation of Population Structure

Information for 211,781 SNPs was obtained from various web sites^3^,^4^; see Cao et al., 2011; Horton et al., 2012) and was used to analyze population structure and for the GWAS.

Population structure among *Arabidopsis* accessions was estimated using an admixture model following Pritchard et al. (2000) with the model-based program STRUCTURE v. 2.3.4^5^ and a set of 1000 selected SNPs. Selection of the 1000 SNPs was based on the following criteria: (1) MAF ≥ 10%, (2) no missing calls for all accessions, (3) consisting of two alleles, and (4) having similar intervals. STRUCTURE was used to estimate the number of subpopulations [defined as L(K), where K is the number of ancestor subpopulations inputted] and the Q-matrix (indicating ancestor subpopulation components of each accession by given K) for K = 1 to 15. The burn-in period was set to 50,000, with the Markov Chain Monte Carlo iterations and run length set to five replications of 50,000. The largest possible number of K (i.e., 6), which was used for genome-wide association study (GWAS), was determined by the ΔK method (Evanno et al., 2005) using the formula L′(K) = L(K) – L(K–1), | L′(K)| = | L′(K + 1) – L′(K)|.

### GWAS and Other Genetic Analyses

The GWAS was performed with a compressed linear mixed model using “Q-matrix” + “kinship-matrix” (Yu et al., 2006; Zhang et al., 2010) with the software TASSEL v. 3.0 (Bradbury et al., 2007). The Q-matrix was computed using STRUCTURE, and the kinship-matrix was processed using TASSEL. A total of 175,324 genome-wide SNPs (MAF ≥ 5%, missing call rate ≤ 5%) were used for the GWAS analysis. Genomic prediction (GP) analysis using the glmnet R package (Friedman et al., 2010) was performed to evaluate the cumulative effect of loci linked to the top-ranked SNPs obtained on the basis of *p*-values in the GWAS as previously described (Kobayashi et al., 2016). Using randomly selected SNPs throughout the genome as a reference, the cumulative effects of the linked loci were estimated with 20–300 (each 20 intervals) top-SNPs and defined 140 and 160 top-SNPs as significantly associated SNPs for Al and proton tolerance, respectively. Missing SNPs were imputed using the program BEAGLE (Browning and Browning, 2009). The cumulative effect and predictive accuracies were estimated using 100 replicates of five-fold cross-validation using the coefficient of determination (r^2^) and RMSE respectively as indexes.

The local LD (pairwise r^2^ > 0.80) of each associated SNP was analyzed using other surrounding SNPs within the 10 kb window using the program PLINK v. 1.07^6^; Purcell et al., 2007). Physical positions of the SNPs on the genome, open reading frames (ORF), and untranslated regions (UTR) were obtained from the TAIR 9 database^7^. The genomic DNA region of each gene was defined as the region consisting of a UTR, ORF, and putative promoter (−2 kb from the end of 5′ UTR). Together with the above information, genes located within the LD region of each associated SNP were grouped as the tolerance candidate genes. However, when no LD region was detected in an associated SNP, we chose the closest gene as the candidate associated with the corresponding SNP.

The accessions with unusual phenotype were inferred from the rate of difference in RRL between that observed and that predicted by GP. The difference rate was calculated using the formula log_2_ (observed RRL/predicted RRL). The predicted RRLs were calculated using the average of the RRLs of 100 cross-validations with the top 140 and 160 SNPs detected by the GWAS. The unusual accessions were mapped onto a world map using the “Geocoding and Mapping” web tool^8^. The map of soil pH in Europe was obtained from the European Soil Data Centre’s (ESDAC) ‘Map of Soil pH in Europe’ (Land Resources Management Unit, Institute for Environment & Sustainability, European Commission Joint Research Centre, 2010^9^.

### Reverse Genetics and Co-expression Network Analysis

Al and proton tolerance of T-DNA and mutant lines were judged using the RRL from hydroponically grown seedlings as described in the preceding sections. Co-expression network analyses were conducted using the tool NetworkDrawer implemented in ATTED-II (Obayashi et al., 2018) using co-expression data of “Ath-r” with the ‘add many genes’ option.

### Expression Analysis of Accessions and Expression GWAS

Approximately 100 seedlings of each accession were grown hydroponically for 10 d in control solution (0 μM Al, pH 5.6). Subsequently, the roots were treated with the Al stress solution (10 μM Al, pH 5.0) for 9 h, and the total RNA isolation from the roots and reverse transcription were conducted using Sepasol-RNA I Super G (Nacalai Tesque, Kyoto, Japan) with High-Salt Solution for Precipitation (Plant) (Takara Bio, Japan) and ReverTra Ace qPCR RT Master Mix with gDNA Remover (Toyobo, Osaka, Japan), respectively, following the manufacturer’s instructions. Quantitative RT-PCR was performed using the standard curve as previously described by Bustin et al. (2009). *AtSAND* (AT2G28390) was used as an internal control, and the gene expression level of each accession was normalized by that of Col-0 as the control of experimental batches. Sequences of gene-specific primers used for qPCR are shown in the Supplementary Table S3. Gene expression level of each accession was defined by mean from three replicates. The *p*-values for the correlation between the SNP alleles (MAF ≥ 10%) and gene expression levels were calculated from the expression level of accessions with the tolerant and sensitive allele (from 8 to 17 biological replicates per allele) using the “lm” function in R version 3.3.0^10^. Expression GWAS analysis using gene expression level of approximately 70 accessions was conducted in the program TASSEL v. 3.0 using a generalized linear model (GLM) with the genome-wide SNPs used for the GWAS evaluating RRL.

### Sequence Analysis of *AtALMT1* Locus

The *AtALMT1* promoter sequences (-2235 bp from ATG) of *Arabidopsis* accessions were sequenced using direct sequencing for the genomic PCR-amplicons using a BigDye Terminator v. 3.1 Cycle Sequencing Kit (Applied Biosystems), according to the manufacturer’s recommended protocol. Genomic PCR for direct sequencing was conducted using TaKaRa Ex Taq (Takara), and clean-up of PCR products was conducted using ExoSap-IT (Affymetrix). Assembly and multiple sequence alignment were carried out using the programs GENETYX v. 11 (Genetyx) and MEGA 6.06 (Tamura et al., 2013). The *AtALMT1* promoter sequences of *Arabidopsis* accessions determined in this study were submitted to the DDBJ database. The DDBJ accession numbers are shown in the Supplementary Table S8. The sequences of reference accessions (e.g., Col-0) were obtained from the TAIR 10 database. The haplotypes of the *AtALMT1* promoter were initially estimated from the sequence data of 46 accessions with MAF > 10% and haplotype frequency > 10%. We then determined a series of variants constituting the haplotypes for an additional 25 accessions to estimate the four major haplotypes (Supplementary Table S4). The haplotype network of the *AtALMT1* promoter was constructed using the reduced median network method (Bandelt et al., 1995) with the “frequency > 1” criterion in the program NETWORK 5.0^11^. Insertion and deletion sites, including putative transposon element insertions, were handled as a single mutation in the calculation. The accessions with Hap2 type *AtALMT1* promoter were mapped onto a world map as described above.

### GUS Staining, Expression Level Analysis

GUS staining of 5-day-old seedlings was performed following Kosugi et al. (1990) following 9 h of exposure to hydroponic solution containing 10 μM AlCl_3_ at pH 5.0. Expression level analysis of GUS was conducted as described above using *UBQ1* (AT3G52590) as an internal control (three technical replicates in three individual transgenic lines for each construct).

### Malate Excretion Analysis

Malate excretion from the *Arabidopsis* roots was analyzed as previously described (Kobayashi et al., 2007). Approximately 10 seedlings were hydroponically pre-grown for 4 d in sterile growth MGRL medium (pH 5.0) in Magenta GA-7 boxes (Sigma-Aldrich). Subsequently, their roots were aseptically transferred to 2% MGRL medium supplemented with 1% sucrose, with or without 10 μM AlCl_3_ at pH 5.0 in 6-well plates. Root exudates were collected after 9 h, and malate levels were quantified enzymatically using the procedure reported by Hampp et al. (1984). Mean values of three biological replicates in each condition were calculated.

## Results

### Variation of Al and Proton Tolerance Among *Arabidopsis* Accessions and Subpopulations

**FIGURE 1:**
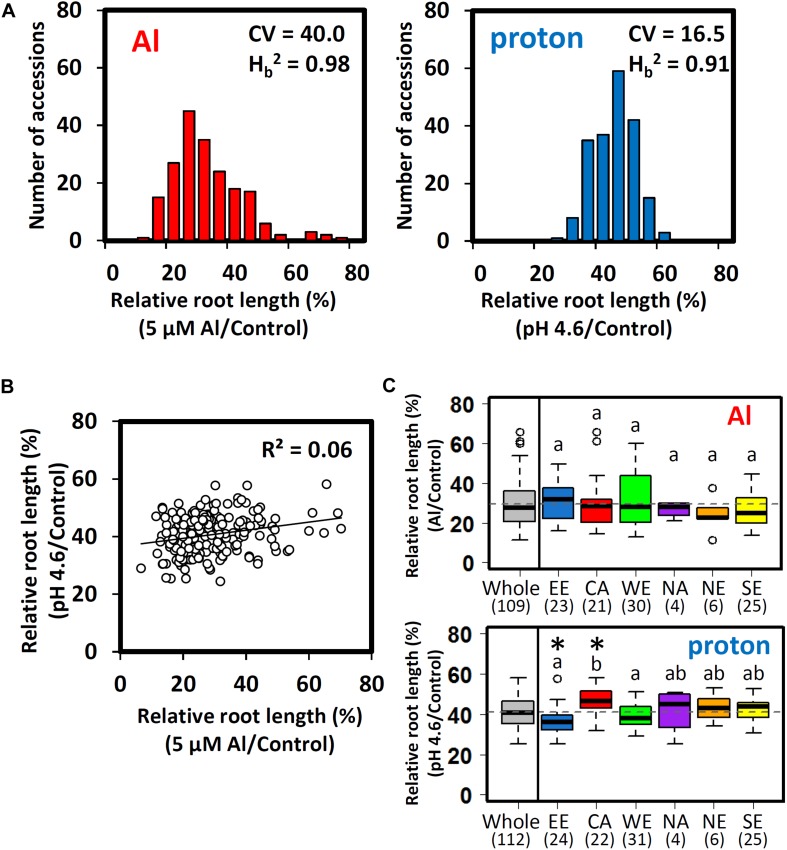
Al and proton tolerance of *Arabidopsis thaliana* accessions. **(A)** Distribution of the relative root lengths (RRLs) of 206 *A. thaliana* accessions under Al and proton stress conditions (CV; coefficient of variation, H_b_^2^; broad-sense heritability). Seedlings were grown hydroponically for 5 days in either Al (5 μM Al, pH 5.0)/proton (0 μM Al, pH 4.6) solutions or a control solution (0 μM Al, pH 5.0). Five biological replicates of root length were used for calculation of relative root length [RRL; root length under stress conditions/root length under control conditions (%)] (*n* = 5). **(B)** Correlation between Al and proton tolerance among *A. thaliana* accessions. **(C)** Boxplot of Al and proton tolerance for 112 representative accessions from six ancestral subpopulations inferred from STRUCTURE (EE; Eastern Europe, NA; North America, CA; Central Asia, WE; Western Europe, NE; Northern Europe, SE; Southern Europe). The values under EE-SE represent the number of representative accessions of each subpopulation (Supplementary Table S1). Significant outliers from the mean RRL for each subpopulation are indicated by open circles above or below the boxplots. The mean RRL value for the whole population is represented by a dashed line. Asterisks above the boxplots indicate a significant difference from the mean RRL value for the whole population (permutation test, *p* < 0.05). Different letters indicate statistically significant differences in mean RRL value among the six subpopulations (Tukey’s HSD test, *p* < 0.05).

The relative root length of seedlings grown in Al (RRL_Al_; pH 5.0 plus 5 μM Al to minus Al) and proton (RRL_proton_; pH 4.6 to pH 5.0) hydroponic culture correlates with the tolerance of *Arabidopsis* to Al (Kobayashi et al., 2005) and proton (i.e., proton stress, Kobayashi et al., 2013) rhizotoxicities in acid soils. We scored the indices of 206 accessions of *Arabidopsis thaliana* from the 1001 Genomes Project collection (see Seren et al., 2017), which included subpopulations adapted to multiple geographic locations. Using the STRUCTURE software, we identified six ancestral subpopulations (Supplementary Figure S1), which can be grouped by the geographic distribution of accessions, corresponding to six locations [Eastern Europe (EE), North America (NA), Central Asia (CA), Western Europe (WE), Northern Europe (NE), and Southern Europe (SE) (Supplementary Table S1 and Supplementary Figure S1)]. After excluding accessions with a low germination rate (*n* ≤ 5), we obtained 196 RRL_Al_ and 200 RRL_proton_, respectively (Figure 1A). While broad-sense heritability estimates were similar (*H*_b_^2^_Al_ = 0.98, *H*_b_^2^_proton_ = 0.91), variation in Al tolerance responses was more than twice as large as that for proton tolerance responses, as estimated by the coefficient of variation (CV) of each phenotype (CV_Al_ = 40.0, CV_proton_ = 16.5). However, there were no significant correlations between Al and proton tolerance among the accessions (Figure 1B). These results suggested that, for the most part, each trait is differently regulated and segregated among *Arabidopsis* accessions.

Differences in segregation patterns between Al and proton tolerances among subpopulations were compared using 112 accessions (i.e., accessions without typical admixture of subpopulations), which carried more than 70% of the estimated membership of each ancestral subpopulation (Supplementary Table S1). There were no significant differences in the mean RRL_Al_ between subpopulations. However, there were significant differences in the mean RRL_proton_ between the CA (proton tolerance) and the EE subpopulations (proton sensitive) (permutation test, *p* < 0.05; Figure 1C). Several subpopulations showed larger within-subpopulation variation of RRL_Al_ (WE and EE) and RRL_proton_ (NA). However, these subpopulations showed relatively lower levels of within-subpopulation variation for the other trait. These observations suggested that Al and proton tolerance did not co-segregate between and within the subpopulations. Several accessions were significantly more tolerant or sensitive in comparison to other accessions belonging to the same subpopulation. Only one accession of the EE subpopulation showed unusual proton tolerance (RRL_proton_), whereas four accessions of the CA and NE subpopulations showed remarkable differences in Al tolerance (RRL_Al_) when compared to other members of the same subpopulation (Figure 1C). This suggests that the unusual phenotype of Al tolerance may occur more frequently than for proton tolerance.

### Identification of Effective Loci That Control Al and Proton Tolerance

**FIGURE 2:**
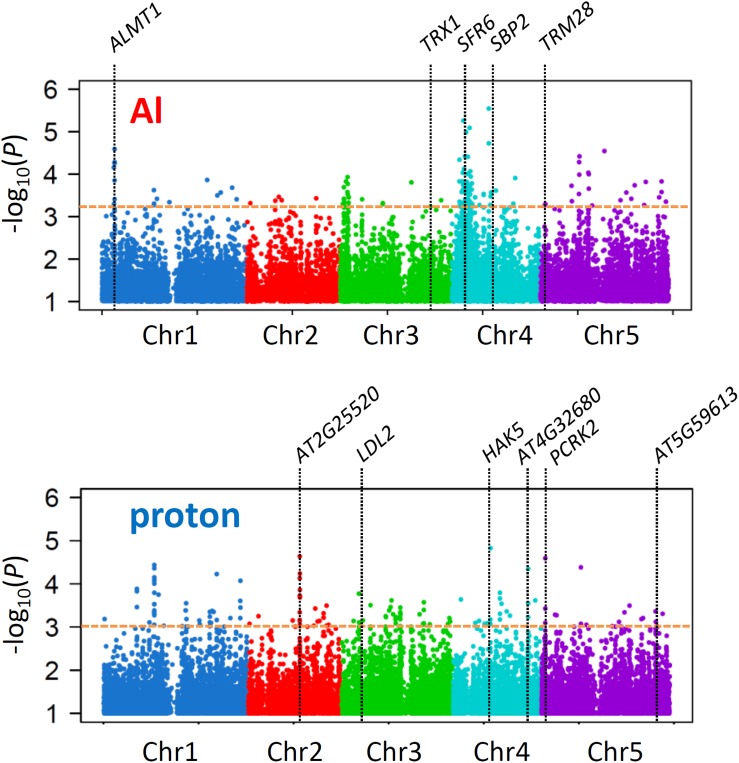
Association mapping for Al and proton tolerance. Manhattan plots for GWA mapping of Al and proton tolerance. Only SNPs with a *p*-value < 0.1 are shown. The horizontal dashed line indicates the *p-*value cutoff based on the cumulative effects of the associated SNPs (Al; *p* < 6.2 × 10^–4^, proton; 1.1 × 10^–3^) respectively. The loci of the genes are shown as a part of genes influenced on the tolerances (Figure 5).

GWA mapping using linear mixed models in the TASSEL software (Bradbury et al., 2007), utilizing 175,324 genome-wide SNPs (MAF ≥ 5%, missing call rate ≤ 5%), identified several loci controlling each trait. The different shapes of Manhattan plots obtained by GWA mapping suggested that our analyses successfully identified different loci controlling Al and proton tolerance variations (Figure 2). Ridge regression analyses of the phenotype (RRL_Al_ and RRL_proton_) and genotype of accessions (i.e., genomic prediction; GP) were conducted using the top-ranked SNPs (i.e., SNPs with the lowest *p*-value in GWA mapping; Figure 2) to estimate effective SNPs, which in relatively small numbers cumulatively explain large proportions of phenotypes.

**FIGURE 3:**
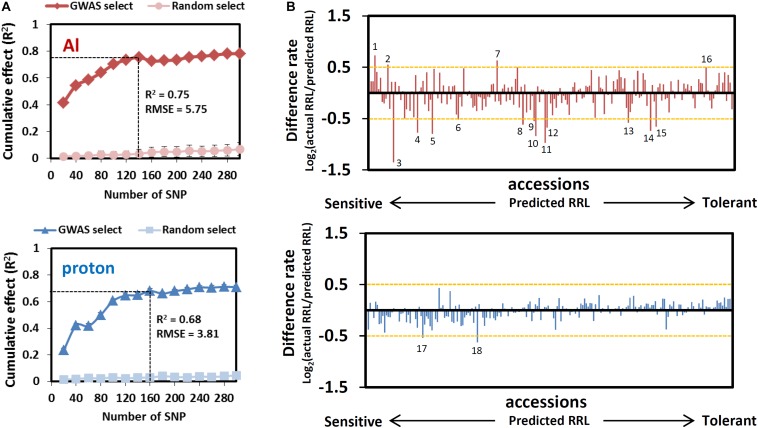
Estimation of the cumulative effect of GWAS detected SNPs by genomic prediction. **(A)** Cumulative effect of 20–300 top-ranked SNPs detected by the GWAS of Al and proton tolerance. Cumulative effects and prediction accuracies were calculated using 100 times 5-fold cross-validations using coefficients of determination (r^2^) and root mean squared error (RMSE) as indexes respectively. **(B)** Difference rate of Al and proton tolerance between actual RRL and predicted RRL. The orange horizontal dashed line indicates the cutoff for unusual phenotype (|Difference rate| ≥ 0.5). Numbers on or under bar plot represent the accessions showing an unusual phenotype (1: Lm-2, 2: Tsu-0, 3: Voeran-1, 4: Bil-7, 5: Bak-2, 6: Dra-2, 7: Fei-0, 8: Bolin-1, 9: Lp2-2, 10: Pna-17, 11: Nermut-1, 12: Bch-4, 13: Jablo-1, 14: Pa-3, 15: Ct-1, 16: Shigu-2, 17: Dra-2, 18: Buckhorn Pass).

Both *R*^2^ curves of the GP, which indicate the proportion of the phenotype explained using a given number of SNPs, peaked before they attained plateau (Figure 3A). According to the corresponding number of SNPs that presented the highest *R*^2^ values before plateauing, we assumed that the 140 (Al) and 160 (proton) SNPs would most effectively explain each trait with relatively small numbers. The highest *p*-values for these SNPs determined by GWA mapping were less than 6.2 × 10^–4^ for RRL_Al_ and 1.1 × 10^–3^ for RRL_proton_ (see Figure 2), and each set of SNPs explained approximately 75 and 68% of phenotypic variation of each trait, respectively. None of the associated SNPs was detected in both GWA mapping analyses, which reinforces the observation that Al and proton tolerance were unrelated (Figure 2). The *R*^2^ of the ridge regression of GP for the top-20 SNPs was greater for Al tolerance (approximately 40%) than for proton tolerance (approximately 20%) (Figure 3A). This suggests that a larger proportion of RRL_Al_ is controlled by a relatively small number of loci in comparison to the proportion of RRL_proton_.

The RMSE (root-mean-square error) in GP evaluates the difference between predicted phenotype and observed phenotype for all accessions. Al tolerance (at 120 SNPs, RMSE = 5.75) had a larger RMSE than proton tolerance (at 140 SNPs = 3.81) which suggests that individual accessions show larger differences between predicted and observed RRLs under Al stressed conditions. To test this, we calculated the “difference rate” [i.e., Log_2_(observed RRL/predicted RRL)] of individual accessions under Al and proton toxic conditions (Figure 3B). Although most accessions showed small differences (difference rate < |0.5|) between the predicted and observed RRL in both conditions (Figure 3B), 12 and four accessions showed markedly different observed RRL from predicted RRL in Al and proton tolerance, respectively (indicated in Supplementary Table S1). This observation suggests that rare-allelic mutations, or other genetic events that induce unusual phenotypes, may occur more frequently in Al tolerance than in proton tolerance.

**FIGURE 4:**
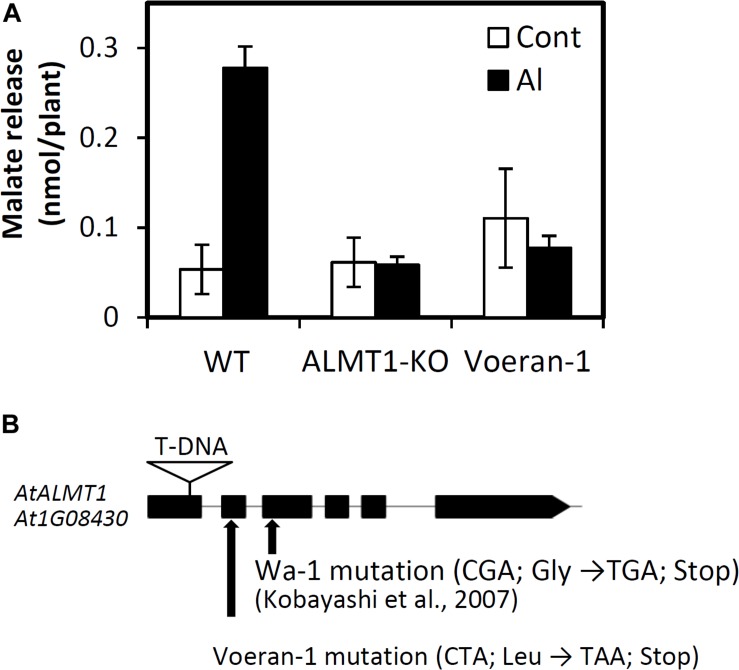
Characterization of Al hypersensitivity in Voeran-1. **(A)** Malate excretion from the root of WT (Col-0), knockout mutant of AtALMT1 and Voeran-1 under control (0 μM Al, pH 5.0) and Al (10 μM Al, pH 5.0) condition for 9 h. Mean values ± SD are shown (average of three biological replicates samples; *n* = 3). **(B)** Position of mutation in *AtALMT1* of Voeran-1 and Wa-1, and T-DNA inserted site of knock out mutant.

Among the unusual accessions, Voeran-1 showed the largest difference in its RRL_Al_ when compared using GP (Difference rate = -1.35; 6.6% in observed RRL and 16.9% in predicted RRL). The accession showed no Al inducible malate excretion, which was comparable with the *AtALMT1*-knockout (KO) mutant (Figure 4A). We confirmed that a mutation introducing a premature STOP codon was present in *AtALMT1* of Voeran-1 by sequencing (Figure 4B), which explains why its hypersensitivity to Al stress deviated from the GP.

### Identification of Genes Control Al and Proton Tolerance Associated With Effective SNPs

We identified total 453 and 578 candidate gene that were located within the 10 kb region (average linkage disequilibrium [LD] decay of *Arabidopsis*; Kim et al., 2007) flanking the 140 and 160 GWAS-detected SNPs for Al and proton tolerance, respectively. The genes listed in Supplementary Tables S5, S6 were investigated for their contribution to the observed phenotypic variation, in order to identify mechanisms underlying the natural variation detected by GWA mapping. The gene list contained some reported tolerance genes such as *AtALMT1*, which has been previously associated with Al tolerance (Hoekenga et al., 2006). However, most genes had never been reported as controlling Al or proton tolerance. The contribution of these unidentified genes were evaluated by reverse-genetics and co-expression gene network analysis.

**FIGURE 5:**
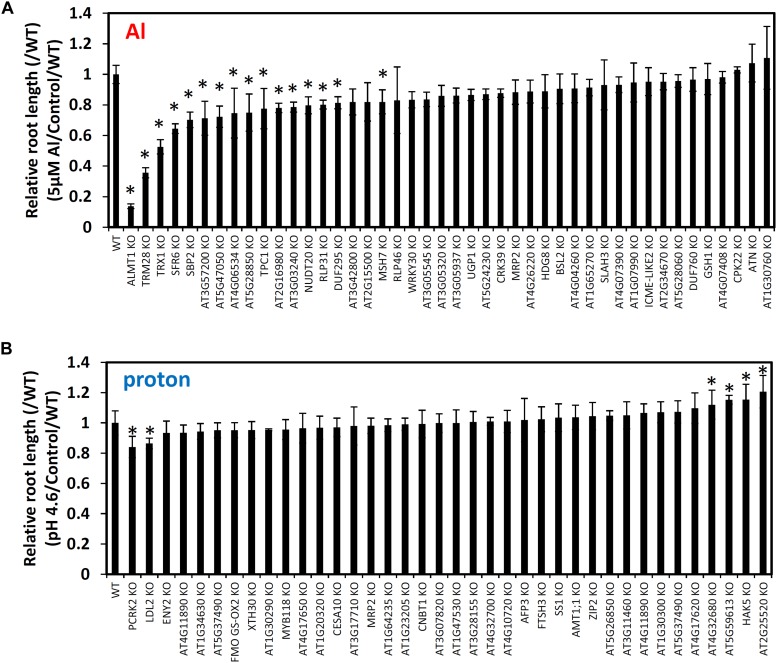
Root growth of mutant lines of GWAS-identified candidate genes for Al **(A)** and proton **(B)** tolerance. Seedlings were grown hydroponically for 5 days in either Al (5 μM Al, pH 5.0)/proton (0 μM Al, pH 4.6) solutions or a control solution (0 μM Al, pH 5.0). Five biological replicates of root length were used for calculation of relative root length (RRL; root length under stress conditions/root length under control conditions). It was divided by the RRL value of WT. Mean values ± SD are shown (*n* = 5). Asterisks indicate significant differences (*p* < 0.05; Student’s *t*-test) compared to WT.

The genes in the list were first filtered as to whether or not they were in the local linkage disequilibrium (LD) block (r^2^ ≥ 0.8), with the detected SNPs calculated individually (i.e., 168 genes for RRL_Al_ GWA mapping and 187 genes for RRL_proton_ GWA mapping; Supplementary Tables S5, S6). Reverse-genetics approaches were applied for all the publicly available mutants of the filtered genes at world-wide *Arabidopsis* bioresource centers (i.e., 44 and 38 genes of the genes detected by Al and proton GWA mapping). Using this approach, we found that 16 and 6 mutants showed significantly altered RRL_Al_ and RRL_proton_ tolerance respectively (Student’s t-test, *p* < 0.05) (Figure 5).

The most sensitive of these knockouts was the previously studied *AtALMT1* (RRL [/WT] = 0.14), which served as a positive control for our analyses. However, all of the other 16 mutants with reduced tolerance to Al stress were newly identified by this study, these include: *TON1 RECRUITING MOTIF 28* (*AtTRM28*; *At5G03670*) (RRL [/WT] = 0.36), *SENSITIVE TO FREEZING 6* (*AtSFR6*; *AT4G04920*) (RRL [/WT] = 0.53) and *THIOREDOXIN H-TYPE 1* (*AtTRX1*; *AT3G51030*) (RRL [/WT] = 0.64) (Figure 5). Mutant analysis for proton tolerance led to decreased stress tolerance far less frequently; in fact, only two of the six knockouts had reduced tolerance to proton stress while the other four were more tolerant. The mutants of *LSD1-LIKE2* (*AtLDL2;* AT3G13682) and *PATTERN-TRIGGERED IMMUNITY COMPROMISED RECEPTOR-LIKE CYTOPLASMIC KINASE 2* (*AtPCRK2;* AT5G03320) were mildly more sensitive to proton stress, while the mutants of *HIGH AFFINITY K^+^ TRANSPORTER 5* (*AtHAK5*; AT4G13420), three genes encoding drug/metabolite transporter superfamily protein (AT2G25520), ATP synthase (AT5G59613) and transmembrane protein (AT4G32680) were mildly more tolerant to proton stress. These results suggest that we successfully identified several genes that control natural variation of Al and proton tolerance in *Arabidopsis* polygenically.

**FIGURE 6:**
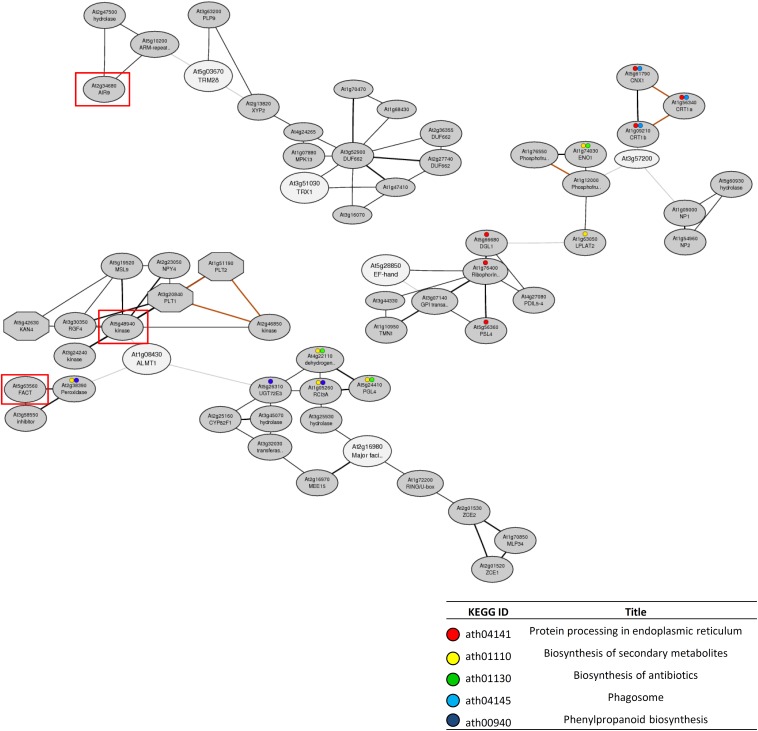
Co-expression networks containing the candidate genes for Al tolerance detected by GWAS. The co-expression networks were constructed by using 16 candidate genes that showed altered Al tolerance in the mutant (represented on Figure 5) as query genes by ATTED-II. Among all constructed networks (Supplementary Figure S2), only the networks that contained more than two candidate genes are shown. White and gray ellipses indicate query genes and added co-expressed genes respectively. Red rectangle indicates the genes located within the 10 kb of GWAS-detected SNP for Al tolerance (Supplementary Table S5) respectively. Colored circles indicated the genes involved in enriched biological processes represented on the table.

Co-expression gene network analysis was conducted using the ATTED-II database to identify additional tolerance genes to those found using the reverse genetics approach. Although no networks were formed by proton tolerance genes, we found three co-expression gene networks that contained multiple Al tolerance genes identified by reverse-genetics (Supplementary Figures S2, S3). In addition, each network contained several other genes that were linked to the effective SNPs for RRL_Al_ by GP (Figure 6). One co-expression network contained *AtALMT1* and AT2G16980. This network was composed of the two Al tolerance genes and 25 co-expression genes including two GWAS-detected genes (*FATTY ALCOHOL:CAFFEOYL-COA CAFFEOYL TRANSFERASE* [*FACT*] and *RGF1 INSENSITIVE 2* [*RGFR2;* AT5G48940]) located within ± 10 kb of the 30^th^ and 41^st^ associated SNPs respectively. Another network was composed of *AtTRM28* and *AtTRX1*, which were demonstrated to have a relatively large contribution to Al tolerance (Figure 5), and 14 co-expression genes including one GWAS-detected gene *AUXIN-INDUCED IN ROOT CULTURES 9* (*AIR9*) located within ± 10 kb of the 79^th^ associated SNP. Among the three co-expression networks, two networks contained genes involved in biological processes including “protein processing in endoplasmic reticulum” and “biosynthesis of secondary metabolites.” These biological processes are regulated by each network and may have important roles in Al tolerance.

### Expression Level Polymorphism of *AtALMT1* and *AtTRX1*

**FIGURE 7:**
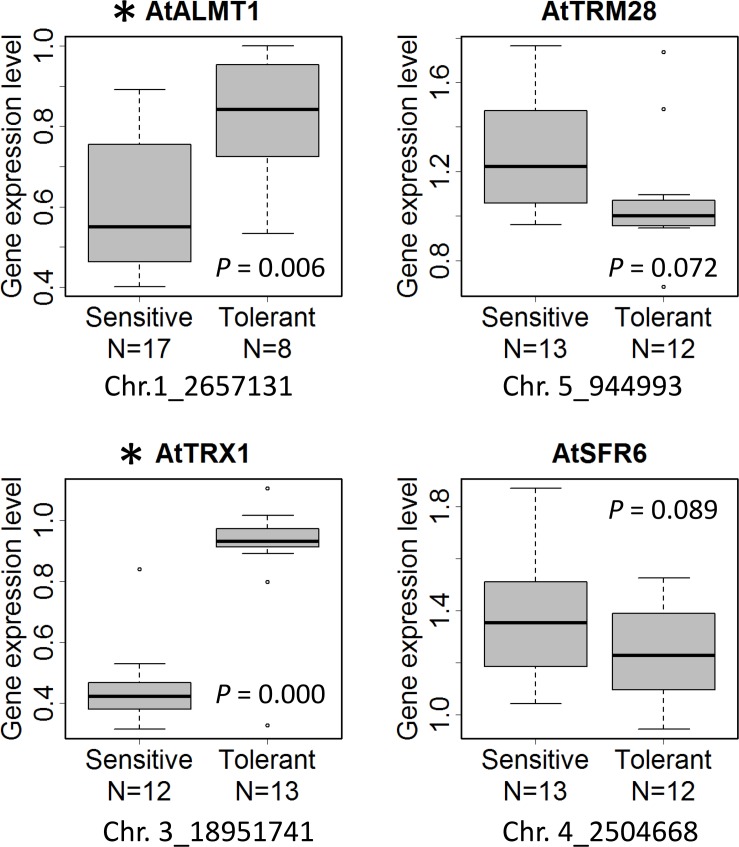
Box plot of expression level of the genes that showed more than 30% decrease of Al tolerance in the mutants (Figure 5). The gene expression level of the accessions carrying sensitive and tolerant allele of the most associated SNP linked with the gene in GWAS (Supplementary Table S5) are shown. Asterisk indicates a significant difference of expression level between the accessions with tolerant and sensitive alleles (*p* < 0.05). N indicates the number of biological replicates per allele (*n* = 8–17).

Expression level polymorphism is one of the mechanisms which causes phenotypic variation of Al tolerance among *Arabidopsis* accessions (Kusunoki et al., 2017). Using randomly chosen 25 accessions, we measured the expression level of four GWAS-detected Al tolerance genes, *AtALMT1*, *AtTRM28*, *AtTRX1*, and *AtSFR6*, that showed more than 30% decrease of Al tolerance in the mutant compared to WT as shown in Figure 5, and analyzed the correlation between the ELP of tolerance genes and RRL_Al_-associated SNP (Figure 7). We found that expression levels of *AtALMT1* and *AtTRX1* were significantly greater in accessions carrying tolerant allele than in accessions carrying sensitive allele. Both *AtALMT1* and *AtTRX1* are directly linked to top-ranked SNPs, but there was no association between the SNPs and amino acid polymorphisms of either proteins obtained from the 1001 proteomes database (Joshi et al., 2012) (Supplementary Table S7). This suggests that protein polymorphism does not play an important role in the variation in Al tolerance caused by these genes. Instead, it suggests that ELP of *AtALMT1* and *AtTRX1* is involved in the mechanism of RRL_Al_ variation.

**FIGURE 8:**
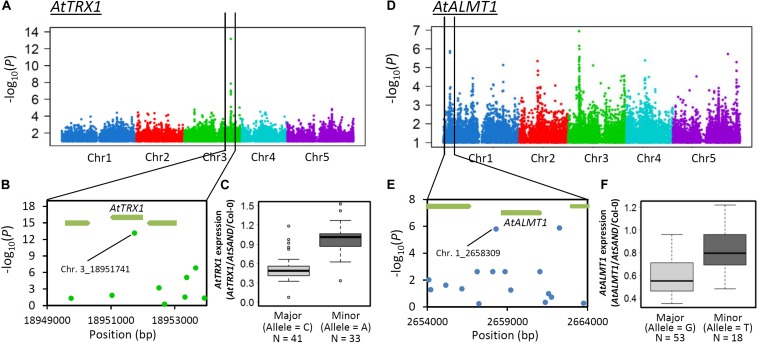
GWA mapping of gene expression level of *AtTRX1* and *AtALMT1*under Al stress. **(A)** Manhattan plots of GWAS of *AtTRX1* expression level. **(B)** Detailed plot of the region of the most associated SNP on Chr.3 in GWAS of *AtTRX1* expression level. **(C)** Box plot of *AtTRX1* expression levels of the most strongly associated SNP (Chr.3_18951741). **(D)** Manhattan plots of GWAS of *AtALMT1* expression levels. **(E)** Detailed plot of the region of higher associated SNP on *AtALMT1* locus in GWAS of *AtALMT1* expression level. **(F)** Box plot of *AtALMT1* expression levels of the higher associated SNP in the *AtALMT1* promoter region (Chr.1_2658309).

Expression GWAS (eGWAS) was conducted on both genes to identify possible mechanisms controlling ELP. The eGWAS of *AtTRX1* solely identified a single peak at its own locus and the most significant SNP was the same as that detected using GWAS of RRL_Al_ (Chr.3_18951741, Figures 8A–C). This strongly suggests that ELP of *AtTRX1*, caused by cis-polymorphism (e.g., polymorphism in promoter), contributes to generating Al tolerance variation. By contrast, eGWAS of *AtALMT1* linked to the *AtALMT1* promoter region and several other loci, suggesting that a portion of ELP of *AtALMT1* could be explained by the difference in promoter activity, which may be directly regulated by the locus (Figures 8D–F). To test this possibility, we conducted haplotype analysis and promoter-*GUS* fusion analysis on *AtALMT1* promoter.

**FIGURE 9:**
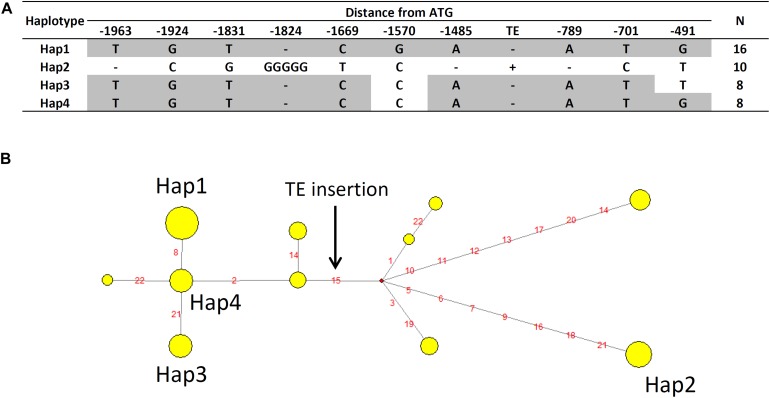
Haplotype analysis of *AtALMT1* promoter. **(A)** Major haplotypes of the *AtALMT1* promoter region observed among 71 *A. thaliana* accessions. The positionsindicate distance from the start codon of *AtALMT1*. “-” indicates deletion. The SNPs of -1669 and -701 correspond to the higher associated SNPs (Chr.1_2657131 and Chr.1_2658099) in GWAS of Al tolerance (Figures 2, 7). The SNP of -491 corresponds to the higher associated SNP (Chr.1_2658309) in eGWAS of *AtALMT1* expression level (Figures 8E,F). **(B)** Haplotype network of *AtALMT1* promoter. Yellow circles represent each haplotype, and circle sizes represent the number of accessions within the haplotype. Red circles represent the median vector. Red letters indicate the variants, and the black arrow indicates the TE insertion.

**FIGURE 10:**
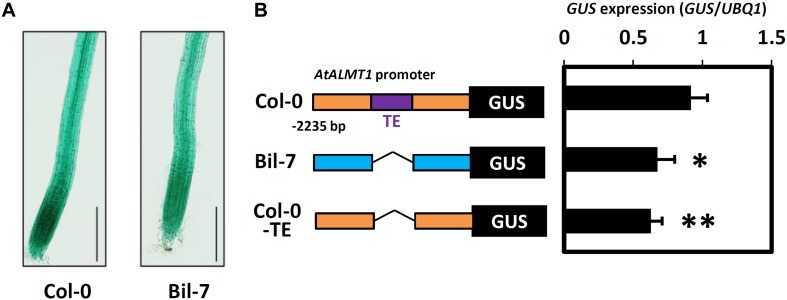
Promoter activity analysis of the *AtALMT1* promoter. **(A)** GUS staining patterns in root of the transgenic plants carrying the *AtALMT1* promoter of Col-0 and Bil-7. Transgenic plants were grown hydroponically for 5-days in a control solution (0 μM Al, pH 5.0) and then exposed to an Al stress solution (10 μM Al, pH 5.0) for 9 h. Bar = 200 μm. **(B)** *GUS* expression levels in the roots of transgenic plants carrying different *AtALMT1* promoters. Col-0 type and Bil-7 type promoters are indicated by orange and blue boxes respectively. TE (498 bp length transposable element insertion at 879 bp upstream from the ATG codon of *AtALMT1*) is indicated by a purple box. *GUS* expression levels were analyzed using real-time quantitative RT-PCR. Approximately 100 seedlings were grown hydroponically for 10 days in a control solution (0 μM Al, pH 5.6) and then treated with an Al stress (10 μM Al, pH 5.0) solution for 9 h. Mean values ± SD are shown (three technical replicates in three individual transgenic lines for each construct). Asterisks indicate significant differences compared with the Col-0 type promoter (Student’s *t*-test, ^∗∗^*p* < 0.05, ^∗^*p* < 0.1).

Haplotype analysis of *AtALMT1* promoter was conducted using 71 accessions. This analysis provided several haplotypes, in which there were four major haplotypes (Hap1-Hap4, frequency > 10%) (Figure 9A and Supplementary Table S4). All 10 accessions with minor alleles of GWAS-detected SNP constituted Hap2, which carried 498 bp insertion corresponding to a transposable element (TE) AT1TE08660 of the ATLANTYS3 family 879 bp upstream of the ORF. Additionally, several SNPs and small indels constituting each haplotype were found (Figures 9A,B). To evaluate this model further, we compared the activity of the Hap2 type promoter (Col-0) and a sensitive promoter (Bil-7) using transgenic carrying promoter-*GUS* (Figure 10). The part of GUS activity in the root was similar in both lines (Figure 10A), however, the *GUS* expression level of the Col-0 promoter-*GUS* line was significantly greater than that of the Bil-7 promoter-*GUS* line (Figure 10B). By contrast, deletion of TE showed lower *GUS* expression compared with that of the Col-0 promoter-*GUS* line (Figure 10B), indicating that greater expression of Hap2 is, in part, caused by the TE insertion, which is involved in the greater expression level of *AtALMT1* observed in Col-0.

## Discussion

Tolerance to Al and proton toxicities are mostly quantitative traits but single major genes can account for a large proportion of the phenotypic variation in many species (e.g., Kobayashi and Koyama, 2002; Hoekenga et al., 2003; Kobayashi et al., 2005; Ikka et al., 2007). In the present study, a GWAS of Al and proton tolerance using the RRLs of *Arabidopsis* accessions identified various genes linked to the detected SNPs, which cumulatively explained approximately 70% of the phenotypic variations of each trait, which included some of major genes controlling each trait (Figure 3A). The identified genes (168 and 187 genes by RRL_Al_ and RRL_proton_ respectively, Supplementary Tables S5, S6) included a number of critical genes (e.g., *AtALMT1* for Al tolerance, Hoekenga et al., 2006) for which dysfunctional mutation could directly alter tolerance (Figure 5). GWAS revealed the cumulative effects of multiple genes that controlled these tolerances, which belonged to the distinct biological process of either Al or proton tolerance (Figure 6 and Supplementary Figures S2, S3). These results provide new insights into the complex mechanisms underlying Al and proton tolerance in plants.

A comprehensive reverse genetics approach using the T-DNA mutants of the GWAS-detected genes revealed the importance of STOP1-regulated genes, *AtALMT1* and *HAK5*, for variation in Al tolerance and proton tolerance, respectively (Figure 5). *AtALMT1*, which encodes an Al activated malate transporter, is one of the critical Al tolerance genes in *Arabidopsis* (Hoekenga et al., 2006) and was linked to the major QTL of the L*er*/Col (Kobayashi and Koyama, 2002) and L*er*/Cvi population (Kobayashi et al., 2005). The T-DNA KO line of *HAK5*, which encodes a high-affinity K^+^ transporter, slightly enhanced proton tolerance (Figure 5). In contrast, higher expression of *HAK5* was observed in the proton-sensitive STOP1 mutant compared to WT when under proton stress (Sawaki et al., 2009). This could account for the role of K^+^ homeostasis in the protection of cells against proton stress through the maintenance of cytosolic pH (Britto and Kronzucker, 2005; Bissoli et al., 2012).

Our GWAS and GP did not identify the STOP1 locus in either Al or proton stress tolerance (Supplementary Table S5, S6). It appears that the gradual adaptation of *Arabidopsis* to acid soils relied on modifications to the genes downstream of this major transcription factor, rather than changes to the transcription factor itself. In contrast, the polymorphism of STOP1-like protein (rice ortholog ART1) was identified as being important for variation in Al tolerance in rice (Arbelaez et al., 2017). This suggests that polymorphisms in STOP1 do not cause the variation in Al and proton tolerance among *Arabidopsis* accessions, where this is not the case in rice. This may be a result of the pleiotropic nature of STOP1-like proteins and the differences in the number of copies in the two species. Rice contains at least five copies of STOP1-like proteins (Yamaji et al., 2009). However, *Arabidopsis* contains only two copies of the genes for STOP1-like proteins (including the STOP1’s downstream STOP2; Kobayashi et al., 2014). Recent studies have identified that dysfunction of STOP1 can repress salt and hypoxia tolerance, while enhancing drought tolerance in *Arabidopsis* (Enomoto et al., 2019; Sadhukhan et al., 2019). This suggests that the polymorphism of STOP1 directly interferes with other stress tolerant traits in *Arabidopsis*, but not in rice, as a result of its redundancy. This hypothesis warrants investigation by further studies.

Our approach, namely integration of GWAS and reverse genetics, would fail to identify several critical genes for Al and proton tolerance due to underlying technical limitations. For example, our GWAS did not detect several critical Al tolerance genes of *Arabidopsis*, such as genes for citrate transporting MATE (Liu et al., 2012) and ALS3 (Larsen et al., 2004), and any genes encoding proteins belong to cell-wall metabolism, while several polysaccharides of cell-wall are involved in Al tolerance mechanisms (Yang et al., 2008). It could be explained by insufficient power of our GWAS conducted with multiple subpopulations to detect the subpopulation specific allele (Korte and Farlow, 2013; Imamura et al., 2016), which may segregate only in some subpopulations. By contrast, the background accession of most T-DNA inserted plants (i.e., Col-0), is one of the most proton sensitive among all accessions (RRL_proton_ = 34.9). It may affect sensitivity of reverse genetic analysis, which evaluate the loss of proton tolerance by the disruption of particular gene. Different approach such as overexpression of GWAS-identified genes in Col-0 would be useful to evaluate the candidate genes for proton tolerance.

Combining GWAS and genome-wide functional genomics approaches, such as joint genetic and network analysis (Kobayashi et al., 2016; Butardo et al., 2017), is a useful approach to elucidate the polygene-regulated tolerance mechanisms. In this study, co-expression gene network analysis revealed that multiple Al tolerance genes identified by reverse-genetics belonged to the same/small co-expression network (Figure 6). The network formed with *AtALMT1* contained another Al tolerance gene and two genes collocated near the top-ranked SNPs of RRL_Al_. The linked genes contained *RGFR2*, which is directly associated with *AtALMT1* in the co-expression network and is a critical protein kinase for root meristem growth (Shinohara et al., 2016). Another network was formed by AtTRX1 and AtTRM28, which showed severe Al sensitivity through the growth assay of T-DNA insertion mutants next to the *AtALMT1*-KO (Figure 5). TRXs play roles in processes that maintain ROS-status and ROS-signaling (Foyer and Noctor, 2005; Navrot et al., 2007; Skelly et al., 2016), while the TRM family proteins are known to regulate polymerization of microtubules, such as the formation of the microtubule array during cell division (Struk and Dhonukshe, 2014; Schaefer et al., 2017). This suggests that AtTRX1 and AtTRM28 may contribute to Al tolerance through the regulation of processes that require microtubules, including cell wall synthesis (Höfte and Voxeur, 2017), which is a typical target biological process by Al (Sasaki et al., 1997; Sivaguru et al., 2003). The network also contained a gene linked to the top-ranked SNPs, which encoded one of the microtubule associated proteins (AT2G34680). The other network, which contained four genes annotated as “protein processing in endoplasmic reticulum (ER),” was formed by three genes which were identified by reverse-genetic assay, and one other gene linked to the top-ranked SNPs (Figures 5, 6 and Supplementary Table S5). This suggests that protein processing in the ER is involved in *Arabidopsis* Al tolerance mechanisms. In fact, a gene encoding the ER-localized protein chaperon (BINDING PROTEIN3) was previously identified as one of the Al tolerant genes in *Arabidopsis* (Kusunoki et al., 2017).

Expression level polymorphism of *AtALMT1* and *AtTRX1* due to cis-regulatory allelic variation was identified as one of the causes of Al tolerance variation detected by GWA mapping. An eGWAS for *AtTRX1* revealed a single and significant linkage of the *AtTRX1* locus, suggesting that greater expression of the gene was mostly determined by mutations in the cis-acting factor, which was also associated with Al tolerance (Figures 7, 8A,B; Supplementary Table S5). This variation is similar to the natural variations in *NIP1*;*1* that regulate H_2_O_2_ tolerance by ELP as a result of mutations in the promoter (Sadhukhan et al., 2017). In this study, mutations in the promoter were also identified as an ELP mechanism of *AtALMT1* (Figures 7, 8D,E). The typical pyramiding of historical mutations consists of TE insertion followed by a single nucleotide mutation (Figures 9A,B). The insertion of a TE is one of the major mechanisms causing ELP that drives adaptation to new environments (Yang et al., 2013). This accounts for the greater expression levels of major Al tolerance genes occurring in several Al tolerant crop varieties such as barley, wheat, sorghum, and rice (Magalhaes et al., 2007; Fujii et al., 2012; Tovkach et al., 2013; Yokosho et al., 2016; Pereira and Ryan, 2019). In this study, most of the accessions with a Hap2 type *AtALMT1* promoter (higher *AtALMT1* expression type) originated from the Western Europe region where acid soils are dominant (Supplementary Figure S4). This suggests that they have adapted to the acid soil common to the region by enhancing their *AtALMT1* expression level. On the other hand, the eGWAS of *AtALMT1* identified complex regulation of *AtALMT1* expression, which has also been identified by previous studies investigating *AtALMT1* expression (e.g., Tokizawa et al., 2015). Further study of the loci detected by eGWAS may uncover the molecular mechanisms which act in this complex system.

Accessions with unusual phenotypes, which were indicated by a large gap between the observed and predicted phenotype using GP, occurred more frequently in Al tolerant accessions (Figures 1A, 3B). By contrast, only few accessions showed unusual phenotypes in proton tolerance (Figures 1A, 3B), supporting our hypothesis that proton tolerance appears to be strongly regulated by polygenes. These differential patterns in the genetic architecture of Al and proton tolerance variation need to be considered when breeding crop varieties tolerant to acid soils. On the other hand, distribution of Al and proton tolerant accessions shows different pattern. Accessions that were unusually tolerant to Al tended to be located in the acid soils regions of Western Europe (Supplementary Figure S4). By contrast, accessions that were unusually sensitive to Al were located in the non-acid soil region of Southern Europe included the most Al sensitive accession, Voeran-1, a natural *AtALMT1* loss-of-function mutant, found in Northern Italy (Supplementary Figure S4). This suggests that variation in Al tolerance would be beneficial in order to adapt to acid soil conditions. However, the loss of Al tolerance would not have negative effects on survival in a non-acid soil environment. By contrast, there were no such trends in proton tolerance levels of accessions. It may be accounted for the pleiotropic role of proton tolerance, which interfere various other traits such as nutrient acquisition and cell expansion (Shavrukov and Hirai, 2016). This hypothesis warrants investigation by further studies.

## Data Availability Statement

All datasets generated for this study are included in the article/Supplementary Material.

## Author Contributions

YN performed most of the experiments and writing. KK, KT, and YS carried out the expression analysis. SI and MK generated the plant materials and assisted with gene sequencing. KK, OH, and YY discussed the study and assisted in the writing and GWA mapping. YK and HK designed the work and supervised, and wrote and edited the manuscript. All authors approved the manuscript.

## Conflict of Interest

The authors declare that the research was conducted in the absence of any commercial or financial relationships that could be construed as a potential conflict of interest.

## Footnotes

1 http://1001genomes.org/

2 https://www.agr.nagoya-u.ac.jp/∼shinkan/LIA32/index-e.html

3 https://cynin.gmi.oeaw.ac.at/

4 http://1001genomes.org/index.html

5 http://pritchardlab.stanford.edu/structure.html

6 http://pngu.mgh.harvard.edu/purcell/plink/

7 http://www.arabidopsis.org

8 http://ktgis.net/gcode/index.php

9 https://esdac.jrc.ec.europa.eu/content/soil-ph-europe

10 http://www.R-project.org/

11 http://www.fluxus-engineering.com/index.htm
