# Supplementary material for: Genome-Wide Association Study and Genomic Prediction Elucidate the Distinct Genetic Architecture of Aluminum and Proton Tolerance in Arabidopsis thaliana
Source: Front Plant Sci. 2020 Apr 9;11:405. doi: 10.3389/fpls.2020.00405 (PMC7160251; doi:10.3389/fpls.2020.00405)
Supplement: Supplementary file 1 [file Data_Sheet_1.PDF]

Supplementary Figure S1

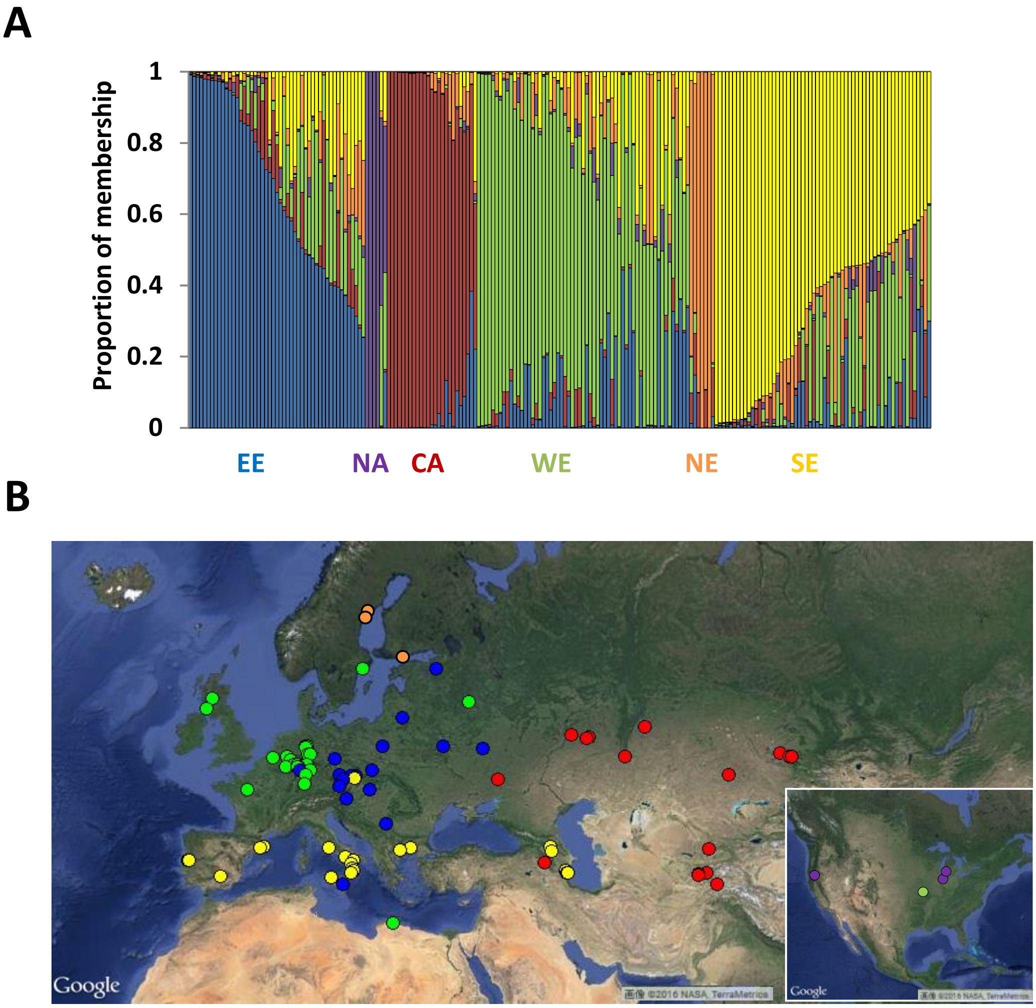

Supplementary Figure S1 Population structure of 206 accessions based on the STRUCTURE result (K = 6). (A) A vertical line represents the proportion of membership of each accession. Each color corresponds to the inferred membership in K = 6 ancestry subpopulations estimated by STRUCTURE (EE; Eastern Europe, NA; North America, CA; Central Asia, WE; Western Europe, NE; Northern Europe, SE; Southern Europe). (B) Geographic distribution of 112 representing accessions, which carried more than 70% of the inferred membership of the particular ancestry subpopulation. Each color corresponds to the ancestry subpopulations represented on (A).

Supplementary Figure S2

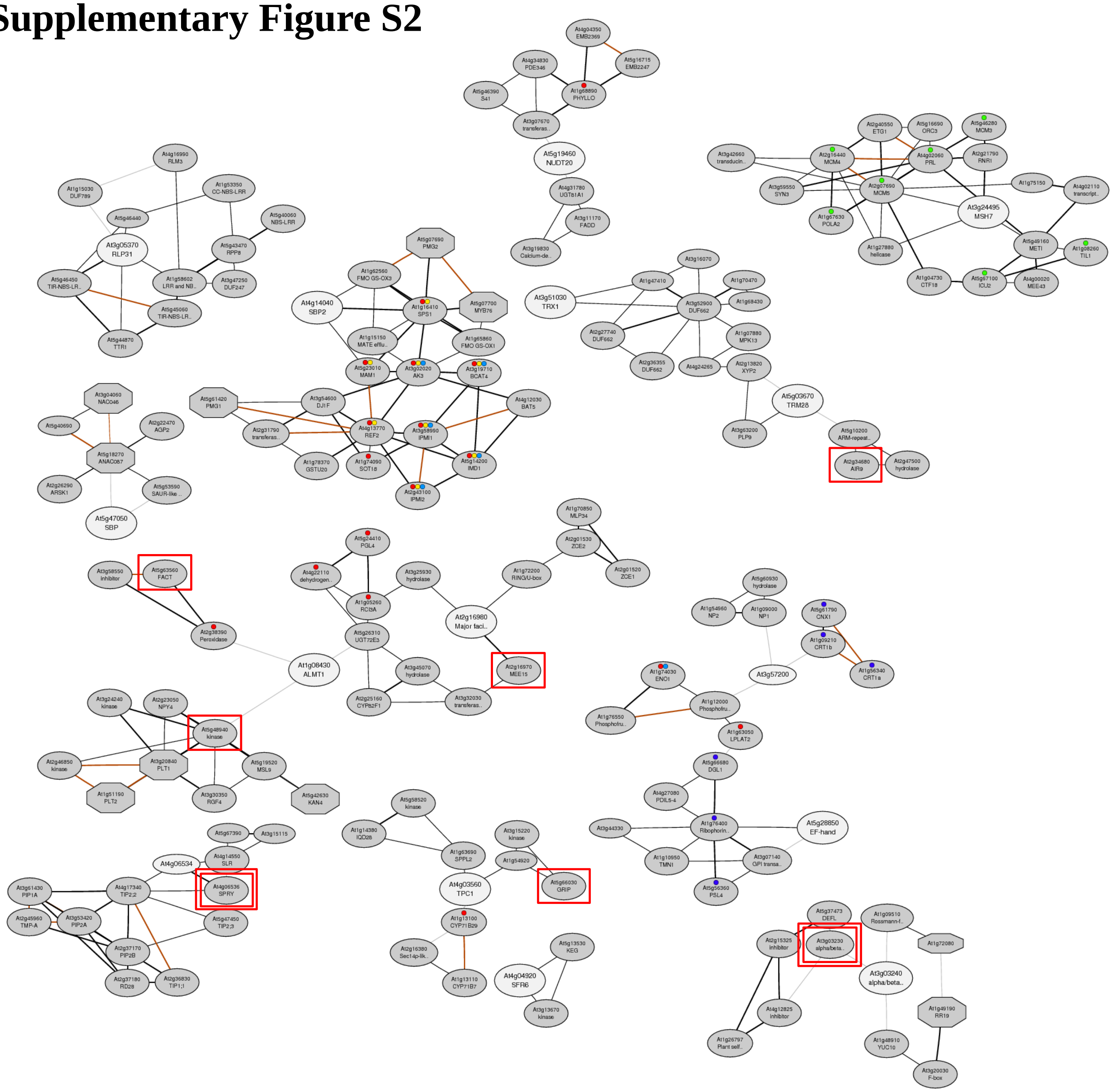

KEGG ID	Title
ath01110 (red)	Biosynthesis of secondary metabolites
ath01210 (yellow)	2-Oxocarboxylic acid metabolism
ath03030 (green)	DNA replication
ath01230 (light blue)	Biosynthesis of amino acids
ath04141 (dark blue)	Protein processing in endoplasmic reticulum

Supplementary Figure S2 Co-expression networks containing the candidate genes for Al tolerance as detected by GWAS. The co-expression networks were constructed using 16 candidate genes that showed altered Al tolerance in loss-of-function mutant (Figure 5) as query genes by ATTED-II. White and grey ellipses indicate query genes and added co-expressed genes respectively. Red single and double rectangles indicate the genes located within the 10 kb and local LD block of GWAS-detected SNP for Al tolerance (Supplementary Table S5) respectively. Colored circles indicate the genes involved in enriched biological processes represented on the table.

Supplementary Figure S3

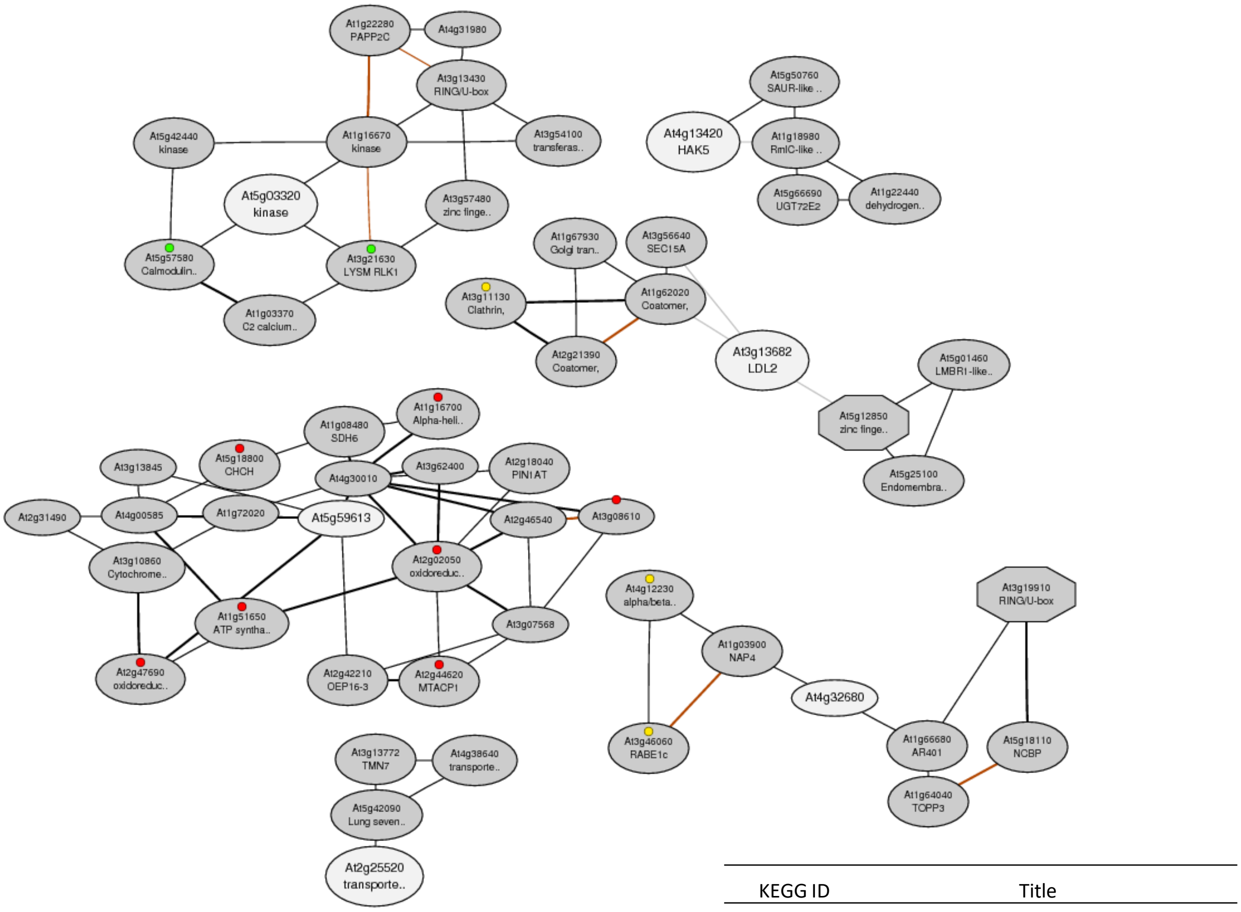

KEGG ID	Title
(red circle) ath00190	Oxidative phosphorylation
(yellow circle) ath04144	Endocytosis
(green circle) ath04626	Plant-pathogen interaction

Supplementary Figure S3 Co-expression networks containing the candidate genes for proton tolerance detected by GWAS. The co-expression networks were constructed using six candidate genes that showed altered proton tolerance in mutant (Figure 5) as query genes by ATTED-II. White and grey ellipses indicate query genes and added co-expressed genes respectively. Colored circles indicate the genes involved in enriched biological processes represented on the table.

Supplementary Figure S4

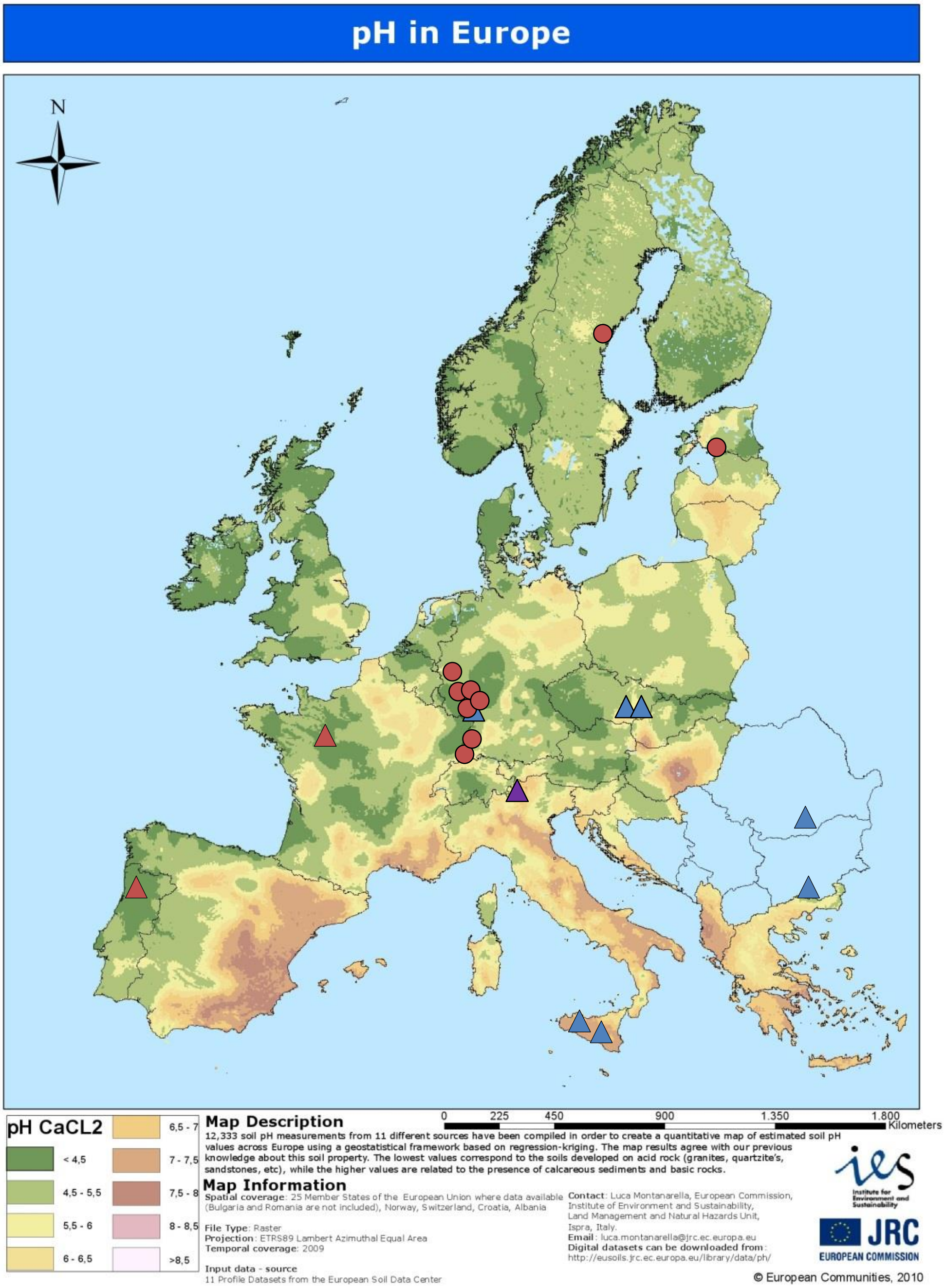

Supplementary Figure S4 Geographic distribution of the accessions with a Hap2 type *AtALMT1* promoter and which showed unusual Al tolerance on the soil pH map of Europe. Red circles represent the accessions with a Hap2 type *AtALMT1* promoter (Figure 9). Red and blue triangles represent the accessions which showed markedly higher and lower Al tolerance than predicted by GP respectively (Figure 3B). The purple triangle represents Voeran-1, which is a natural loss-of-function mutant of *AtALMT1* (Figure 4). The map (Map of Soil pH in Europe) was provided by the European Soil Data Centre (ESDAC; Land Resources Management Unit, Institute for Environment & Sustainability, European Commission, Joint Research Centre, 2010). Green areas indicate more proton regions than red areas.
